# Effects of Foot Structure Type on Core Stability in University Athletes

**DOI:** 10.3390/life13071487

**Published:** 2023-06-30

**Authors:** Orlando Santiago Moreno-Barriga, Carlos Romero-Morales, Ricardo Becerro-de-Bengoa-Vallejo, Marta Elena Losa-Iglesias, Juan Gómez-Salgado, Julio Caballero-López, Liz Carol Vidal-Valverde, Daniel López-López

**Affiliations:** 1 Faculty of Sport Sciences, Universidad Europea de Madrid, 28670 Madrid, Spain; orlandosantiagomorenob@hotmail.com (O.S.M.-B.); juliocabalope@hotmail.om (J.C.-L.); di_carolina_12@hotmail.com (L.C.V.-V.); 2 Department of Nursing, Faculty of Nursing, Physiotherapy and Podiatry, Universidad Complutense de Madrid, 28040 Madrid, Spain; ribebeva@enf.ucm.es; 3 Faculty of Health Sciences, Universidad Rey Juan Carlos, 28922 Alcorcon, Spain; marta.losa@urjc.es; 4 Department of Sociology, Social Work and Public Health, Faculty of Labour Sciences, University of Huelva, 21007 Huelva, Spain; juan.gomez@denf.uhu.es; 5 Safety and Health Postgraduate Programme, Universidad Espíritu Santo, Guayaquil 092301, Ecuador; 6 Research, Health, and Podiatry Group, Department of Health Sciences, Faculty of Nursing and Podiatry, Industrial Campus of Ferrol, Universidade da Coruña, 15403 Ferrol, Spain; daniellopez@udc.es

**Keywords:** foot, public health, stability

## Abstract

Purpose: This study assessed the impact of different types of medial foot arch on postural stability and core center of gravity muscle activity among collegiate athletes. Methods: The study sample included 103 university-level athletes across various sports (soccer, rugby, basketball, volleyball, field tennis, table tennis, karate, and cheerleading) from the College of Magdalena (Colombia) who exhibited distinct types of medial foot arch: 32 high, 35 low, and 36 neutral arches. Surface electromyography (sEMG) was employed to assess conduction velocity, magnitude values, latency, and fatigue in focal muscles including the spinal erector (SE), internal oblique (IO), external oblique (EO), and rectus abdominis (AR), while measurements of static and dynamic postural control were also considered. Post hoc analysis was performed with Bonferroni correction for all electromyographically measured muscle groups, as well as for measurements of static and dynamic postural stability. Pearson’s or Spearman’s correlation tests were used to compare the different types of feet. Results: There were no substantial differences observed between the distinct types of feet in terms of focal muscle activity, static stability, or dynamics. Even though the mean values indicated higher muscle activity and stability among those with high foot arches and lower values among those with low arches compared to the neutral foot type, this observed difference was deemed statistically insignificant. We also observed a positive correlation between internal oblique muscle activity and the average power of dynamic postural stability, which remained consistent across all foot types. Our findings indicate that static instability is directly correlated with dynamic instability in the anteroposterior direction, while a clear inverse relationship was established in the lateral direction upon examining the variable correlations. Conclusions: The presence of high or low foot arches did not significantly impact the activity of the muscles responsible for maintaining the body’s center of gravity or postural stability among university-level athletes. This suggests the existence of neuromuscular compensation mechanisms that attempt to restore balance and compensate for any changes in postural stability caused by varying foot types. Through targeted training that emphasizes activation of the internal oblique muscle, athletes may see improved postural stability. Our findings indicate that static stabilization exercises can also prove beneficial in improving dynamic stability in the anteroposterior plane, while a more dynamic approach may be required to improve dynamic stability in the lateral plane.

## 1. Introduction

Effective stability control is paramount in all sporting activities. The strength, range of motion, and neuro-muscular requirements of the lower limbs play a crucial role in achieving optimal stability, and injuries affecting the base of support can significantly impact athletic performance [1]. Under normal circumstances, feet transition seamlessly between pronation and supination, adapting as required for optimal adaptability versus stability. However, foot misalignments can significantly impact mobility and impair proper function of the lower leg during the stance phase. Excessive pronation or supination can also alter peripheral somatosensory input and adversely affect joint mobility or surface contact area, leading to unstable support in athletes.

Overall, the research underscores the critical role of stability control in sports, placing significant emphasis on the importance of factors such as lower limb strength, range of motion, and neuromuscular demands. Additionally, the research highlights the detrimental effects of foot misalignments, as well as excessive pronation or supination, which can have a marked negative impact on athletes’ mobility and overall performance.

Individuals exhibiting postural changes in their feet experience dynamic body postural control issues due to differences in mechanical stability rather than changes in proprioceptive and neuromuscular function [2].

Achieving adequate body balance necessitates a sound base of support in the feet, which must work in conjunction with the central and peripheral nervous systems to maintain proper alignment and center of gravity. The foot’s arch architecture, as well as its intricate external and internal musculature, play a decisive role in providing stability and acting as a “central core” for the foot [3]. To fully understand the impact of foot structure on pronation (related to a flat or low medial arch) or supination (related to a high medial arch) and its potential to affect postural balance and athletic performance, it is critical to measure both static and dynamic postural stability across each foot type [4].

The various foot types, including flat foot (low), straight foot (neutral), and cavus foot (high), are characterized by both structural and functional differences [5]. Identifying an individual’s foot type can offer valuable insights into the strength and functionality of their foot muscles [6]. Structural variations in foot shape, particularly those characterized as low (flat), neutral (straight), or high (cavus), demonstrate significant impacts on foot function. Individuals with flat feet typically display greater midfoot flexibility, resulting in greater absorption of ground reaction forces but diminished stability and foot stiffness, which translates to lower power generation and reduced force transfer efficiency. As a result, greater intrinsic and extrinsic muscle engagement is required during activities such as walking and running. Conversely, those with high-arched feet exhibit more rigid foot structures, affording higher peak force generation and decreased absorption of ground reaction forces, which can increase the shortening velocity of calf muscles. Additionally, high-arched feet generally display greater stability than flat feet [7,8,9].

The literature on foot dynamics has established that the structure of the arch exerts a significant influence on center of pressure (COP) excursion during functional activities such as walking and running. Several studies have explored this relationship, observing clear associations between foot arch structure and COP motion [5,10,11,12,13,14]. Notably, individuals with pes cavus [15] or flat feet [16] display marked differences in COP excursion, ostensibly linked to the unique structural attributes of these foot types. The observed effect of foot arch structure on center of pressure (COP) excursion can have important consequences for balance and gait control, as it may alter the position of the body’s center of mass. Specifically, the supinated foot position, which typically features an elevated plantar arch and increased ankle inversion force, has been linked to reduced mediolateral stability [17]. The achievement of postural stability relies, in part, on the functional and structural alignment of the feet, which, in turn, requires a balance of mobility and stiffness to properly manage gravitational and ground reaction forces across a range of terrains and activities. This involves a degree of flexibility in foot mobility, allowing for appropriate absorption of these forces, while maintaining sufficient stiffness to support standing, walking, and running stability [18].

Engaging in regular sports activities has been closely associated with noteworthy enhancements in postural stability, even when maintaining the usual bipedal stance [19]. Furthermore, recent research has uncovered that postural stability among adolescent athletes can differ depending on their specific sport and gender [20]. However, these studies did not consider the type of foot, which may also have an impact on the postural balance of athletes.

Despite the potential benefits of training on postural stability in athletes, the efficacy of such training may also be influenced by the type of foot presented by the individual. Previous research has highlighted the potential for foot type, specifically flat feet, to significantly impact ankle and foot movement control in athletes, leading to reduced postural balance [21]; similar results were observed in another study conducted on young athletes participating in football, rugby, basketball, and volleyball [22]; however, another study conducted on young athletes found that the flat foot type did not significantly affect the athletes’ balance [23]. Given this discrepancy and the scarcity of research on the topic, it is important to investigate the potential influence of foot type on athletes’ overall stability.

The impact of foot type on athlete balance is a controversial topic with inconclusive results from previous studies. Even with specialized training, athletes may or may not experience significant changes in their balance. By adding to the existing body of knowledge in this area, we hope to provide greater clarity and understanding of this important topic.

The recruitment of motor units during exercise is largely influenced by the intensity of the activity. To assess the degree of neuromuscular activation, the amplitude of the surface electromyography (sEMG) signal is often utilized. The sEMG amplitude is typically reported in millivolts or as a percentage of the maximal voluntary isometric contraction (%MVIC). This methodology is commonly employed in the scientific community to evaluate the extent to which motor units are activated during physical activity [24].

The central aim of this investigation is to evaluate the impact of varying structural foot types on both postural stability and the engagement of center of gravity muscles in collegiate athletes, using cutting-edge surface electromyography (sEMG) and motion analysis technology.

## 2. Materials and Methods

### 2.1. Design and Sample

This observational and descriptive study aims to assess the athletic male population at Magdalena University, Colombia, aged 18 years and above, representing a diverse range of sporting activities such as rugby, soccer, basketball, volleyball, field tennis, table tennis, karate, and cheerleading. Through convenient stratified random sampling, 35 participants with a flat medial arch, 32 subjects exhibiting a low arch, and 36 with a “normal” arch structure were identified among the 230 candidates. The athletic participants received targeted training specific to their respective sports, with a training regime consisting of 5 to 6 weekly sessions, each spanning 1 to 2 continuous hours. The training program was meticulously crafted by a seasoned fitness trainer and followed strategic cyclicality, encompassing critical stages such as general physical preparation, specific preparation, pre-competition, and competition. All research participants resided in either Santa Marta or neighboring towns situated within the Magdalena vicinity. Recruitment was primarily conducted within the middle and lower–middle social strata. Eligibility for participation required that individuals had engaged in active sports practice within the past four years. Participants with reported pain or active injury with or without biomechanical alterations of the lower extremities, current pain, or pathology; who were undergoing spinal surgery; who had undergone previous abdominal surgery; who had neural or vestibular disease; or who were suffering from arthritis of the lower extremities were excluded from the analysis. Moreover, to ensure the absence of substances adversely affecting postural control, individuals who had used substances such as alcohol, sedatives, pain relievers, cold meditations, stimulants, etc., within the past 24 h were also excluded. Finally, it was determined not to include any candidates with rigid pronated feet in the analysis. This research endeavor upheld strict ethical standards, in full compliance with the human experimentation guidelines enumerated in the Declaration of Helsinki [25]. Approval for execution of the study was conferred by both the Ethics Department of the Universidad del Magdalena and the Ethics Committee of the European University of Madrid.

### 2.2. Foot Type Assessment

**Figure 1:**
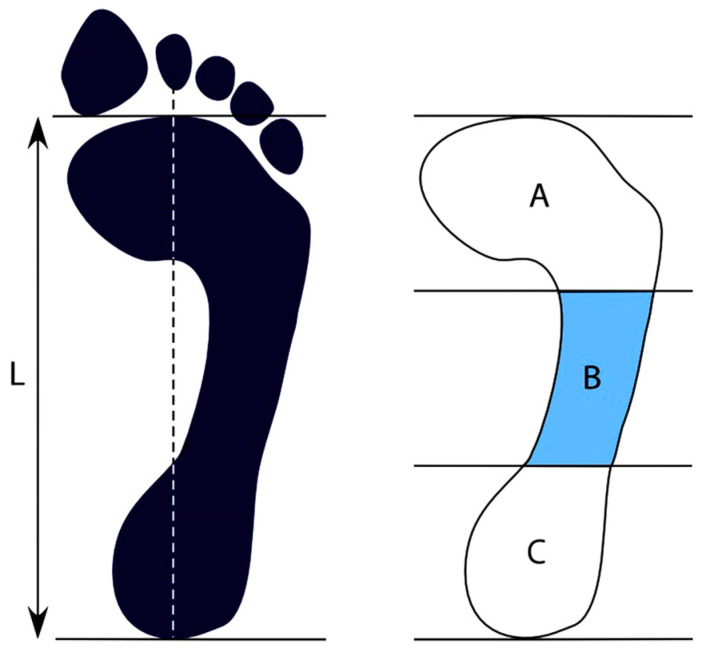
Measurement of the Plantar Arch Index. Source: Beelen et al. (2020) [28]. The arch index (AI) is calculated as: area middle foot (B)/(Total area: anterior (A) + middle (B) + posterior (C)).

The Foot Arch Index serves as the criteria for classifying various types of foot. This measure determines the height of the medial arch of the foot by assessing the ratio between the middle third’s area and the total area of the foot, excluding the toes [26]. An arch index score of less than 0.21 indicates a high or cavus foot, while a score in excess of 0.26 signifies a low or flat foot, with an index ranging from 0.21 to 0.26 reflective of a normal arch [27]. To obtain the plantar footprints of participants, the left foot was coated with a washable ink pad, and the individual was directed to stand on graph paper for one minute while the right foot remained planted on the ground. An Arch of the Foot Index was computed by outlining a vertical line from the midpoint of the heel to the center of the tip of the second toe, identified as Line L in Figure 1. Next, a line perpendicular to Line L was traced tangentially to the lowest point of the print. Line L was then divided into three equal segments perpendicular to it, producing three regions—anterior, middle, and posterior: A, B, and C. AutoCAD® v.14 (AUTODESK, US) software was used to perform the analysis to calculate the arch index, equivalent to B/A + B + C, as per the established protocol [4].

### 2.3. Study Instruments

In this study, static and dynamic postural stability evaluations were conducted using cutting-edge 3D inertial sensor equipment manufactured by GYKO-Microgate^®^ (Bolzano, Italy). Additionally, for dynamic stability assessments, Microgate Optogait^®^ (Bolzano, Italy) equipment was employed. The neuromuscular amplitude of center of gravity muscles was gauged utilizing surface electromyography and electronic myometry, powered by state-of-the-art Biomec^®^ (Biomec Lab, Barcelona, Spain) equipment (Beckman Coulter, Indianapolis, IN, USA). Anthropometric measurements were obtained by means of Bioimpedance equipment InBody^®^ (Biospace, California, USA), thus ensuring the comprehensive evaluation and precise depiction of study participants.

### 2.4. Assessment of the Action of the Core Muscles and Static and Dynamic Stability

This research endeavor employed surface electromyography (sEMG) to obtain muscle activity data concerning the center of gravity. Functional exercises were employed, comprising an unstable Bulgarian squat with weights to target the rectus abdominis (RA), a front plank exercise with scapular adduction and posterior pelvic tilt to engage the internal oblique (IO), a unilateral standing dumbbell press for the external oblique (EO), and a deadlift to activate the spinal erector (SE). Prior studies utilizing electromyography have demonstrated these exercises to be particularly effective in this regard [24]. In accordance with a widely accepted research methodology, the participants in this study undertook the exercises in three separate sets, each lasting for five seconds, and comprising isometric (static) contractions for every muscle group. This method has been widely reported as the most-employed exercise approach for gauging the electromyographic activity of center of gravity muscles [24].

This study adopted the recommended guidelines for electrode placement from established literature sources. Specifically, for the rectus abdominis (RA) muscle, the electrode placement parameters suggested by Garcia-Vaquero et al. [27] were utilized, whereas for the internal oblique (IO), external oblique (EO), and spinal erector (SE), the guidelines outlined by Boccia and Rainoldi [29] were followed.

To accurately gauge static postural control, we employed state-of-the-art 3D equipment capable of measuring the kinematics of body movement. Notably, the test was conducted with participants’ eyes closed, as this has been widely shown to enhance changes in the somatosensory and muscular components of the postural control system. The decision to undertake testing with participants’ eyes closed was purposeful, as previous research has indicated that open eyes may not provide a sufficiently discerning means of accurately evaluating inherent postural control abilities [30]. To ensure consistent and reliable measurements of static postural control, participants in this study were given explicit instructions to maintain the stillest possible posture for a duration of 10 s, while their eyes remained closed. 

To comprehensively evaluate participants’ physical capabilities, this study involved four distinct measurements for the dominant foot, with intervals of 30 s allotted for rest and recovery between each reading. For each measurement, participants were instructed to maintain a hands-on-hips posture, with the non-weight-bearing limb flexed at a precise 45-degree angle. Perhaps most critically, participants were strictly prohibited from either touching the non-weight-bearing limb to the weight-bearing limb, or from placing any part of the non-weight-bearing limb down on the ground. Any such infractions necessitated re-administration of the specific trial in question. Throughout the assessments, we calculated and analyzed total mean distance and speed, anteroposterior mean distance and speed, mediolateral mean distance and speed, as well as covered area. 

To accurately assess dynamic postural control, we employed specialized movement analysis equipment in this study. Specifically, participants were instructed to perform five single-leg jumps using their dominant foot, allowing us to derive the mean power output, as well as left/right and forward/backward displacement and the mean covered area. 

Notably, assessments measuring core muscle strength and both static and dynamic postural control were conducted separately on different days, with static measurements performed prior to dynamic testing to reduce the potential for muscle fatigue to interfere with measurements. Throughout all testing sessions, participants were in a stage of general physical preparation, with no changes made to their dietary intake. Notably, all assessments were conducted in the afternoon, approximately two hours after participants had consumed their main meal of the day. Through this carefully tailored measurement protocol, we were able to gather precise and reliable data regarding participants’ physical capabilities and performance.

### 2.5. Statistic Analysis

In order to conduct in-depth statistical analyses of the gathered data, we employed IBM SPSS Statistics (version 21, IBM Corp., New York, NY, USA). Prior to performing any analyses, we ensured that the data met normality assumptions by utilizing the Shapiro–Wilk test on each variable within each type of foot, which was categorized as low, high, or normal. For quantitative dependent variables such as postural stability and center of gravity muscle activity, ANOVA was utilized when the data followed a normal distribution. When distributions were not normal, the Kruskal–Wallis test was employed.

To establish the homogeneity of variances, we employed Levene’s test. Post hoc analyses, adjusted using the Bonferroni correction, were then used to investigate any significant differences among the groups for each dependent and independent variable (flat, tall, or neutral foot). Furthermore, we utilized Pearson’s correlation to examine the relationships among muscle activity, static and dynamic postural control, and different foot types, when the variables had normal distributions. If the variables were not normally distributed, we relied on Spearman’s correlation to evaluate the potential relationships.

To calculate the simple effect size in individual groups, we utilized ANOVA fixed effects comprising a one-way analysis, as determined using G*Power 3.1.9.7. The test settings were maintained carefully with three independent groups, with α set to 0.05, power (1-β) set to 0.80, and the standardized medium–large effect size (partial eta squared) set to 0.31. Conducting a power analysis allowed us to determine that a total of 105 participants divided equally into three independent groups were required to achieve sufficient statistical power to achieve meaningful results. The significance level was set to *p* ≤ 0.05.

## 3. Results

In Table 1, we present the anthropometric characteristics of university athletes who were divided into low, high, and neutral foot categories. The results obtained by implementing ANOVA or Kruskal–Wallis and post hoc analysis with Bonferroni correction for electromyographic muscle activity, static postural control, and dynamic postural control, are reported in Table 2. Our findings indicate that there were no statistically significant differences in age, muscle mass, height, fat, and muscle percentage when comparing the different foot types among university athletes.

Table 2 provides detailed descriptive values and comparison results for the variables of muscular activity and postural control among the high, low, and neutral foot groups of the athletes. Our analysis revealed that athletes with high foot arches had higher mean values for the variables of muscular activity and static postural control, while those with low foot arches had lower mean values for these variables. Additionally, participants with high foot arches exhibited less area covered for dynamic postural control when compared to those with low foot arches, indicating greater central stability in the former group and less stability in the latter group, in comparison to athletes with the neutral foot type. However, despite these trends, we found that there were no significant differences observed for any of the variables when comparing the high, low, and neutral foot groups.

As we observed no significant differences between the high, low, and neutral foot groups, we sought to establish a relationship between the variables irrespective of foot type by conducting Spearman’s correlation analysis; Table 3 presents these results. We found a positive correlation between the activity of the external oblique muscle and the mean power of dynamic postural control (r = 0.21, *p* < 0.05), indicating that increased activity in the external oblique muscle is associated with greater stability in dynamic postural control. Moreover, we observed positive correlations between the total distance of static postural control and the anteroposterior displacement of dynamic postural control (r = 0.18, *p* < 0.05), and between the anteroposterior distance of static postural control and the anteroposterior displacement of dynamic postural control (r = 0.20, *p* < 0.05), indicating that greater static postural control is associated with better dynamic postural control. Finally, we also identified a negative correlation between the area covered by static postural control and the right/left displacement of dynamic postural control (r = −0.19, *p* < 0.05), suggesting that a larger area covered by static postural control is associated with decreased right/left displacement in dynamic postural control (Table 3).

## 4. Discussion

The aim of the present study was to explore the connection between foot type (low, high, and neutral) and postural stability among university athletes, using surface electromyography (sEMG) and motion analysis technology. Our hypothesis was that there would be an association between foot structure and postural stability. However, our findings provided limited evidence to support this notion. This is consistent with the study by Tudor et al. [23], which also found no significant differences between flat foot type and athlete balance. Our comparison of the various foot types revealed no statistically significant differences in center of gravity muscle among activity and static or dynamic postural stability. Although participants with high arches exhibited slightly better muscle activity and stability, whereas those with low arches showed lower muscle activity and stability compared to individuals with neutral foot structure, the differences were not statistically significant.

The aim of the current study was to explore the correlation between high and supinated feet and body stability in athletes. The results demonstrated that individuals with increased body mass exhibited lower stability. Specifically, athletes with high and supinated feet were found to be more vulnerable to body instability, particularly individuals with higher body mass [28]. However, these athletes showed better postural stability when they had adequate body mass [31]. Interestingly, a reduction in the base of support (for example, a high foot arch) with the absence of vision was found to decrease postural stability in young adults [32]. Conversely, limited evidence of an association between foot type and static stability was found in another study [2]. Athletes with high and supinated feet demonstrated increased dynamic postural stability with open eyes than those with the low foot arch type. However, no significant differences were observed between the two groups when postural stability was measured with the eyes closed.

The primary goal of this scientific inquiry was to examine the relationship between foot type and postural stability among university athletes, as well as the strength of the muscles responsible for maintaining the center of gravity. While some research suggests that high foot arches may have a positive impact on postural stability, this remains an area of inconsistency. Additionally, it is unclear whether these findings are applicable to individuals with normal feet. Our study aimed to delve deeper into these questions and address gaps in the current research.

Our results indicate that foot type is not a significant factor affecting postural stability or muscle strength in university athletes. These findings suggest that “compensatory” mechanisms may be at play, whereby the body makes adjusting movements to stabilize for low- or high-arched feet through neuromuscular processes such as motor control feedback.

It is worth noting that discrepancies in the results of previous studies may be attributed to multiple factors, such as the type of population being studied (healthy individuals vs. athletes), differences in measurement techniques, types of variables measured, and measurement conditions (eyes open vs. closed, single leg vs. bilateral support, use of the dominant foot or not). These factors may help to explain the inconsistent findings on foot type and postural stability.

Further research is necessary to gain a thorough understanding of the mechanisms underlying foot type and postural stability in athletes, and to expand our knowledge of how to improve balance and reduce injury risk in a variety of populations.

The mechanisms that may account for the relationship between foot type and postural stability could be related to “postural anticipatory adjustments” commonly used in posture and movement coordination. These adjustments may help to minimize the changes in postural balance associated with low- or high-arch foot types. Furthermore, compensatory strategies utilized by the ankle and hip muscles, including the buttocks, play a crucial role in postural control [17,33]. Depending on the type of disturbance that the body needs to address, such as predictable, unpredictable, or “deceptive”, different neuromuscular responses, such as proactive, reactive, or “anticipatory”, may be generated [34]. Studies have demonstrated that the specific type of sport an athlete engages in affects their postural balance, likely as a result of adaptive strategies employed in overall balance control that minimize the effects of external perturbations [20]. Therefore, future studies should evaluate the participation of these adjustments in a population with three types of foot, comparing athletes versus sedentary individuals to assess the potential for greater compensatory stimuli in the former.

The current study, as well as previous research, indicates that foot type has no significant effect on postural stability during isolated movements, even in athletic populations that may have developed compensatory mechanisms to maintain stability. It is crucial to note, however, that real-world sporting activities involve multiple lower extremity contractions that can lead to fatigue and subsequently cause a decline in posture and movement execution [32,35]. Therefore, it is important that future studies evaluate the influence of foot type on central stability under conditions of fatigue.

Within our study, we discovered an intriguing finding that was independent of foot type, be it high-arched, low-arched, or neutral. Specifically, we found a positive correlation between the activity of the internal oblique muscle and the average power of dynamic postural stability. Our results suggest that by targeting the internal oblique muscle through a front plank with scapular adduction and a posterior pelvic tilt exercise, there is a modest yet significant improvement in postural stability under movement conditions.

It is noteworthy that our findings align with those of prior research that investigated individuals who had undergone Pilates exercises, which focus on central stabilizing muscles. In these studies, participants experienced increased activity in the internal oblique muscle and a heightened ratio of internal oblique-to-rectus abdominis activity. They also exhibited greater pelvic and trunk stability when compared with those who did not engage in this activity. This outcome conferred a greater range of mobility to the lower extremities and produced enhanced flexion traction of the knee over the hip [36], leading to improved dynamic postural stability. Studies have shown that strengthening the postural stabilizer muscles of the trunk can stimulate the functional muscular “corset” present in the center of the body by increasing the thickness or hypertrophy of the internal oblique and transversus abdominis muscles, which, in turn, improves postural stabilizer motor control. This beneficial impact was particularly observed in children with central hypotonia [37], suggesting that exercises targeting the activation of the internal oblique muscle can lead to significant improvements in dynamic postural stability, even among athletes.

Our study revealed that static instability exhibits a direct correlation with dynamic instability in the anteroposterior direction, while it is inversely related in the lateral (right-left) direction. In a study conducted on obese patients [38], researchers concluded that there was a substantial increase in anteroposterior displacement with both open and closed eyes compared to their non-obese counterparts. This outcome is commonly attributed to the loss of muscle mass in these patients, especially of fast-twitch muscle fibers that are responsible for maintaining postural stability. It can also be due to the alteration of their center of mass (center of gravity), and possibly due to their motor incoordination (related to decreased functional muscle activity). However, these factors would not affect stability in athletes who are not obese, and there must be other factors that influence this correlation. Patients with plantar fasciitis [39] and elderly individuals [40] have also demonstrated a direct relationship between static instability and anteroposterior dynamic instability, both of which are related to changes in strength and muscle tone in the ankle and hip muscles, primarily the flexors of the fingers and abductor hallucis. This relationship is also attributed to the integration between visual, vestibular, and somatosensory systems such as proprioception and skin sensitivity [40,41]. Thus, notable differences in these factors exist among athletes and are dependent on the level of sports practice, the capacity and adaptation to training [42,43], the type of exercise [44], and other relevant factors. All the aforementioned factors collectively underlie the direct correlation between static postural stability and dynamic postural stability in the anteroposterior plane among athletes.

A fascinating study of female jockeys has shown that they exhibit superior stability in the mediolateral axis when measured under unstable conditions, both with open and closed eyes. However, they demonstrated superior control in the anteroposterior axis when assessed under stable conditions [45]. Conversely, a study carried out on female basketball players revealed that the addition of plyometric exercises to their workout routines improved dynamic postural control in the mediolateral plane [46]. This outcome suggests that dynamic training, which produces postural instability, can significantly enhance postural control in the mediolateral direction.

The current study aims to provide clarity on the role played by foot type on the overall balance of athletes. Accordingly, future investigations would do well to comprise a group of non-athletic participants to facilitate a more detailed and rigorous understanding of this influence.

The limitations of the present study stem from the fact that the evaluation was restricted to a particular group of athletes, namely, male university students aged between 18 and 24. Consequently, the outcomes are relevant only to this specific population. Our decision to establish correlations regardless of foot type was motivated by the lack of significance in the post hoc comparisons. Nonetheless, this approach constitutes a limitation to our researc, due to the fact that the applied correlations did not consider foot type within the athletic population. The sample size, too, was deemed inadequate to yield high power, given our selection of an effect size between medium and high (f: 0.31), resulting in a total of 105 participants. Additionally, the nature of the sport engaged in may influence an athlete’s postural balance ability; thus, we propose that future studies analyze postural balance regarding each foot type in designated sports.

## 5. Conclusions

Our study findings suggest that the presence of a high or low foot arch does not markedly impact the muscles associated with maintaining postural stability or center of gravity in university athletes. Nevertheless, the results appear to indicate the existence of neuromuscular compensatory mechanisms that strive to rectify deviations in static and dynamic postural stability in athletes with high and low foot arch types. Given the direct correlation between static postural stability and dynamic stability, particularly on the anteroposterior plane, concentrated static postural training may prove critical to enhancing dynamic stability on this plane. Conversely, exercises integrating dynamic conditions may be necessary to enhance dynamic stability on the lateral plane (left to right). To facilitate a more thorough examination, we strongly recommend incorporating non-athletic individuals as a variable in future studies analyzing the influence of foot type on athlete stability.

## Figures and Tables

**Table 1:** *Descriptive data of the groups with low, normal, and high foot arches*.

	Low Foot Arches	Normal Foot Arches	High Foot Arches	F (ANOVA) or H (Kruskal–Wallis)	*p*-Value
N	35	36	32		
Mean age (years) (SD)	22 ± 3.8	22.2 ± 3.1	21.5 ± 2.88	1.21 *	0.54
Mean mass (kilograms) (SD)	76.2 ± 13.9	75.3 ± 12.4	73.3 ± 9.9	0.64 *	0.72
Mean height (m) (SD)	1.76 ± 7.8	1.76 ± 7.5	1.75 ± 8.1	0.46 *	0.79
Mean % fat (SD)	14.8 ± 6.8	16.3 ± 6.5	14.7 ± 6.6	2.27 *	0.32
Mean % muscle (SD)	47.9 ± 4	47.2 ± 4.2	48.2 ± 4.2	0.66 **	0.51

N: sample (subjects), SD: standard deviation. * significance: Kruskal–Wallis; ** significance: ANOVA.

**Table 2:** Descriptive statistics for muscle activity, static and dynamic postural control, and comparison of results of ANOVA or Kruskal–Wallis between groups.

Mean ± SD ANOVA or Kruskal–Wallis								
	Variable		High (n = 32)	Low (n = 35)	Normal (n = 36)	*F (ANOVA) or H (Kruskal–Wallis)*	*p*	Post hoc Comparisons
Muscle activity by electromyography	Amplitude rectus abdominis (mv)		1.88 ± 1.45	1.25 ± 0.79	1.26 ± 0.96	5.47 **	*0.065*	*No differences*
	Amplitude internal oblique (mv)		1.50 ± 1.07	1.17 ± 0.67	1.35 ± 0.74	1.52 **	*0.46*	*No differences*
	Amplitude external oblique (mv)		1.37 ± 1.01	1.21 ± 0.63	1.30 ± 0.62	0.93 **	*0.62*	*No differences*
	Amplitude spinal erectus (mv)		1.23 ± 0.57	1.10 ± 0.59	1.16 ± 0.61	0.417 *	*0.66*	*No differences*
Static postural control Total distance		Total mean (mm)	51.7 ± 35.4	52.5 ± 34.3	49.9 ± 39.8	0.42 **	*0.8*	*No differences*
		Speed (mm)	120 ± 92	105 ± 61	111 ± 75.6	0.088 **	*0.95*	*No differences*
	Anteroposterior distance	Total mean (mm)	33.6 ± 23	33.8 ± 21.9	32.6 ± 32.1	0.33 **	*0.84*	*No differences*
		Speed (mm)	78.4 ± *63.3*	*67.3* ± *35.4*	*67.2* ± *39.5*	0.34 **	*0.84*	*No differences*
	Medio-lateral distance	Total mean (mm)	32.7 ± 25.5	33.1 ± 28.2	32.4 ± 26	0.76 **	*0.96*	*No differences*
		Speed (mm)	73.8 ± 57.6	66.9 ± 44.2	69.9 ± 58.8	0.1 **	*0.95*	*No differences*
	Covered area total mean (mm^2^)		36,988 ± 60,011	32,717 ± 46,348	31,584 ± 72,966	0.77 **	*0.67*	*No differences*
Dynamic postural control	Mean power		7.68 ± 2.3	9.17 ± 11.1	7.4 ± 2.2	0.71 *	0.49	*No differences*
	Left/right displacement		−0.79 ± 2.25	−0.79 ± 2.25	−0.07 ± 2.2	1.680 *	0.19	*No differences*
	Forward/backward displacement		0.072 ± 1.64	0.044 ± 1.54	−0.210 ± 1.84	0.33 *	0.71	*No differences*
	Covered area		124.6 ± 130.4	139.4 ± 199.2	113.1 ± 125.8	0.052 **	0.97	*No differences*

*Mean* (Ⴟ) and ANOVA, or Kruskal–Wallis; F: * value of ANOVA; H: ** value of Kruskal–Wallis; *p*: significance.

**Table 3:** Significant correlation coefficients for muscle activity and static and dynamic postural control.

Variable		1	2	3	4	5	6	7
Muscular activity	1. IO amplitude					0.21 *		
Static postural control (total mean)	2. Total distance							0.18 *
	3. AP distance							0.20 *
	4. Covered area						−0.19 *	
Dynamic postural control	5. Mean power	0.21 *						
	6. LR displacement				−0.19 *			
	7. FB displacement		0.18 *	0.20 *				

RA: rectus abdominis; IO: internal oblique; EO: external oblique; SE: spinal erector; AP: anterior–posterior; ML: medio-lateral; LR: left/right; FB: forward/backward. * p < 0.05.

## Data Availability

Data will be available under formal request.
